# Supplementary figures and images for: Effect of Yeast Culture on Reproductive Performance, Gut Microbiota, and Milk Composition in Primiparous Sows
Source: Animals (Basel). 2023 Sep 18;13(18):2954. doi: 10.3390/ani13182954 (PMC10525930; doi:10.3390/ani13182954)

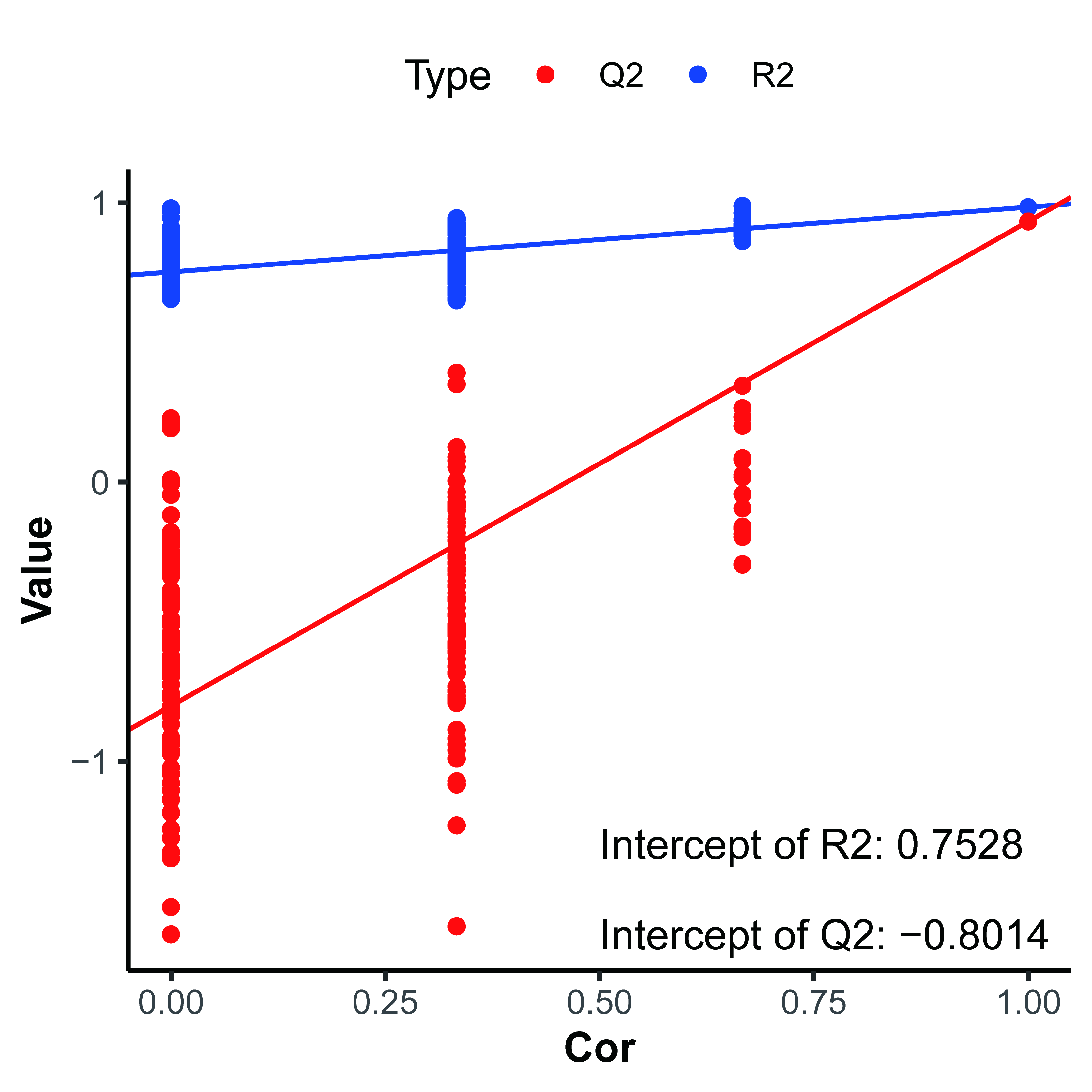

Supplement: Supplementary file 1 [file animals-13-02954-s001.zip › Figure S1.tif]
